# Thomas Carlyle—A Fragment

**Published:** 1893-02

**Authors:** 


### A FRAGMENT FROM THOMAS CARLYLE.

The reminiscences of this distinguished philosopher and author, written sixty years ago, contain the following pathetic words recalling his deceased father: "I shall now no more behold my dear father with these bodily eyes; with him a whole threescore and ten years of the past have doubly died for me. It is as if a new leaf in the great book of time were turned over. Strange time—endless time; of which I see neither end nor beginning. All rushes on. Man follows man. His life is a tale that has been told; yet under Time does there not lie Eternity? Perhaps my father, all that essentially was my father, is even now near me, with me. Both he and I are with God. Perhaps, if it so please God, we shall in some higher state of being meet one another, recognize one another. As it is written, we shall be forever with God. The possibility, nay, in some way the certainty, of perennial existence daily grows plainer to me. 'The essence of whatever was, is, or shall be, even now is.' God is great. God is good. His will be done, for it will be right.

As it is, I can think peaceably of the departed love.

All that was earthly, harsh, sinful in our relation has fallen away; all that was holy in it remains. I can see my dear father's life in some measure as the sunken pillar on which mine was to rise and be built. The waters of time have now swelled up round his (as they will round mine); I can see it all transfigured, though I *touch* it no longer.

I might almost say his spirit seems to have entered into me (so clearly do I discern and love him); I seem to myself only the continuation and second volume of my father. These days that I have spent thinking of him and of his end are the peaceablest, the only Sabbath that I have had in London. One other of the universal destinies of man has overtaken me. Thank heaven, I know, and have known, what it is to be a son; to love a father, as spirit can love a spirit. God give me to live to my father's honor and to His. And now, beloved father, farewell for the last time in this world of shadows! In the world of realities may the Great Father again bring us together in perfect holiness and perfect love! Amen!"

